# Limb-girdle muscular dystrophy type 2B causes HDL-C abnormalities in patients and statin-resistant muscle wasting in dysferlin-deficient mice

**DOI:** 10.1186/s13395-022-00308-6

**Published:** 2022-11-29

**Authors:** Zoe White, Zeren Sun, Elodie Sauge, Dan Cox, Graham Donen, Dmitri Pechkovsky, Volker Straub, Gordon A. Francis, Pascal Bernatchez

**Affiliations:** 1 Department of Anesthesiology, Pharmacology & Therapeutics, University of British Columbia (UBC), 217-2176 Health Sciences Mall, Vancouver, BC V6T 1Z3 Canada; 2 UBC Centre for Heart Lung Innovation, St. Paul’s Hospital, Vancouver, Canada; 3 Newcastle University Translational and Clinical Research Institute, Newcastle upon Tyne, UK; 4 Department of Medicine, UBC, Vancouver, Canada

**Keywords:** Dysferlin, Dyslipidemia, Simvastatin, Cholesterol

## Abstract

Limb-girdle muscular dystrophy (MD) type 2B (LGMD2B) and Duchenne MD (DMD) are caused by mutations to the Dysferlin and Dystrophin genes, respectively. We have recently demonstrated in typically mild dysferlin- and dystrophin-deficient mouse models that increased plasma cholesterol levels severely exacerbate muscle wasting, and that DMD patients display primary dyslipidemia characterized by elevated plasma cholesterol and triglycerides. Herein, we investigate lipoprotein abnormalities in LGMD2B and if statin therapy protects dysferlin-deficient mice (Dysf) from muscle damage. Herein, lipoproteins and liver enzymes from LGMD2B patients and dysferlin-null (Dysf) mice were analyzed. Simvastatin, which exhibits anti-muscle wasting effects in mouse models of DMD and corrects aberrant expression of key markers of lipid metabolism and endogenous cholesterol synthesis, was tested in Dysf mice. Muscle damage and fibrosis were assessed by immunohistochemistry and cholesterol signalling pathways via Western blot. LGMD2B patients show reduced serum high-density lipoprotein cholesterol (HDL-C) levels compared to healthy controls and exhibit a greater prevalence of abnormal total cholesterol (CHOL)/HDL-C ratios despite an absence of liver dysfunction. While Dysf mice presented with reduced CHOL and associated HDL-C and LDL-C-associated fractions, simvastatin treatment did not prevent muscle wasting in quadriceps and triceps muscle groups or correct aberrant low-density lipoprotein receptor (LDLR) and 3-hydroxy-3-methylglutaryl coenzyme A reductase (HMGCR) protein expression. LGMD2B patients present with reduced serum concentrations of HDL-C, a major metabolic comorbidity, and as a result, statin therapy is unlikely to prevent muscle wasting in this population. We propose that like DMD, LGMD2B should be considered as a new type of genetic dyslipidemia.

**Supplementary Information:**

The online version contains supplementary material available at 10.1186/s13395-022-00308-6.

## Introduction

Muscular dystrophies (MD) are a heterogeneous group of muscle wasting disorders. Limb-girdle muscular dystrophy (MD) type 2B (LGMD2B) is caused by mutations to the Dysferlin gene, whereas the more common and severe Duchenne MD (DMD) is caused by mutations to the Dystrophin gene [1]. Dysferlin [2] is a transmembrane protein that regulates calcium-dependent recruitment and fusion of repair vesicles to damaged sarcolemma and trafficking of surface-bound proteins [3]. Loss of normal dysferlin expression or activity causes muscle wasting around early adulthood (10–30 years), ultimately resulting in complete ambulatory dysfunction approximately 10–20 years after diagnosis [2]. In contrast, DMD leads to wheelchair dependency by 12 years of age, due to profound skeletal muscle wasting and associated cardiorespiratory failure [4–6]. Unlike DMD where advances in pharmacological approaches have helped improve life expectancy [7–9], there are currently no treatment options for LGMD2B patients.

Our group has shown using phenotypically mild dysferlin-deficient (Dysf) and dystrophin-deficient (*mdx*) mouse models of LGMD2B and DMD that raising plasma cholesterol via apolipoprotein E (ApoE) inactivation, a key gene involved in lipid metabolism, leads to the drastic exacerbation of muscle disease severity [10]; indeed, Dysf/ApoE double-knockout (DKO) and *mdx*/ApoE double-knockout (DKO) mice display humanized lipid- or fibrosis-rich muscle infiltrates, severe muscle wasting, and clinical ambulatory dysfunction, especially when fed a high-fat diet (HFD) [10], therefore suggesting that dysregulated plasma lipoprotein metabolism likely plays an exacerbating role in MD-associated muscle wasting. We further went on to demonstrate in Dysf and *mdx* mice that muscle disease severity can be titrated to plasma lipoprotein content and more importantly mitigated in both models using cholesterol blocking agents, like ezetimibe [11]. From a clinical perspective, we also reported primary dyslipidemia, including elevated total cholesterol (CHOL) and triglyceride (TG) levels in dystrophin-deficient DMD patients [12–14], unmedicated DMD dogs, and heterozygous female carriers [12]. Recent miRNA profiling of plasma from pediatric DMD patients has also highlighted the dysregulation of genes that govern lipid metabolism and endogenous cholesterol synthesis, including sterol-regulatory binding proteins (SREBP) and downstream mevalonate pathway mediators, such as HMGCR [15]. Two independent laboratories have also reported that the HMGCR inhibitor simvastatin can effectively prevent adverse cardiac, skeletal and diaphragm muscle histopathologies in *mdx* mice [15–17]. While this likely occurs in a pleiotropic fashion, others have stressed that statins are of little therapeutic value in dystrophin-deficient *mdx* mice [18–20], which casted doubts about the true role of endogenous cholesterol synthesis and statin pleiotropism in MD.

To better understand the clinical relevance of abnormal lipid handling in settings of dysferlin deficiency, the present study compared lipid content of LGMD2B patients and observed that, in contrast to DMD, both male and female LGMD2B patients displayed significant reductions in HDL-C levels when compared to controls. While Dysf mice exhibited lower CHOL and associated LDL-C and HDL-C fractions compared to WT mice, their muscle tissues show heightened HMGCR and LDLR protein expression. Despite lowering CHOL, statin pleiotropism had no beneficial effects on histological markers of muscle wasting in Dysf-deficient mice. DMD and LGMD2B may be considered a new heterogenous family of primary genetic dyslipidemias.

## Materials and methods

### Human samples and serology

Serum samples from LGMD2B and age-matched controls were obtained from the MRC Biobank for Rare and Neuromuscular Diseases curated at Newcastle University. The National Research Ethics Service (NRES) Committee Newcastle and North Tyneside 1 approved prior collection of samples from all patients with informed consent for research use (REC number: 19/NE/0028). Age, sex, medication use, and 10-m timed walk test data sets for the LGMD2B patients included in this study were retrospectively obtained from the clinical outcome study for dysferlinopathy (COS) study records. Sera from patients taking cholesterol-lowering drugs (including statins and ezetimibe) or steroids were excluded from selection. Of note, lipoprotein levels are shown to change minimally in response to food intake (triglycerides show the most variation although at maximum 20% or ± 0.3 mmol/L) [21, 22]. Whole blood was centrifuged at 2850 g for 10 min, the supernatant plasma removed, stored at −80 °C pending use and later processed at UBC-affiliated St. Paul’s Hospital (Human Ethics no. H19-01573) on a Siemens Advia 1800 system for CHOL, HDL-C, and triglycerides (TG) [10, 23]. Low-density lipoprotein (LDL-C) cholesterol was calculated using the formula: TC–HDL-C-(TG/2.2). Murine plasma was collected in heparinized tubes via cardiac puncture of anesthetised mice, spun down at 4000 RPM for 10 min at 4 °C, and stored at −80 °C. Samples were processed as mentioned above with minor modifications. Normal plasma lipid levels in adult men and women > 20 years were described as follows: TC (< 5.2 mmol/L), LDL-C (< 3.4 mmol/L), HDL-C (> 1.03 mmol/L in men and >1.3 mmol/L in women), and nonHDL-C (< 4.1 mmol/L) [21, 22, 24]. Given the postprandial effects on circulating TG levels, normal ranges were capped at < 1.7 mmol/L for men and 1.46 mmol/L for women based on comparative studies characterizing fasting and non-fasting plasma TGs in adults [21, 22, 24].

### Animal models and husbandry

All animals were housed in a 12-h/12-h light/dark cycle, temperature-regulated facility. All animal procedures were approved by the UBC Animal Care and Ethics Committees, and all experimental procedures conformed to the Declaration of Helsinki. Experimental male and female Dysf-deficient (Dysf; B6.129-*Dysf*^tm1Kcam^) and wild-type (WT) C57BL/6 mice were obtained by in-house breeding and genotyped [10, 23, 25]. Dysf and C57BL/6 mice were supplemented ad libitum with either a regular chow (CHOW; LabDiet 5001) or a high-fat diet (HFD; Envigo, TD.88137; 0.2% total cholesterol, 21% total fat, and 34% sucrose by weight) in order to replicate both normolipidemic and hyperlipidemic conditions. Dietary interventions were started at 2 months of age. Simvastatin (JAMP Pharma) was formulated and administered in drinking water as previously described [16, 17]. Briefly, 50 mg of simvastatin was dissolved in 1 ml EtoH to activate and solubilize the active (hydroxyl acid) form of simvastatin before adding to 1 L of alkaline drinking water (pH~10) [16, 17]. Mice were treated simvastatin (starting at 2 months of age), and drinking water was changed twice weekly. Untreated mice received normal alkaline drinking water.

### Gait tracking

Analysis of gait was completed from ink imprint of hind paws recorded on paper from mice traversing a 1.5-m distance. Step length was defined as the distance between two consecutive imprints from the same foot. Areas in which a mouse paused were not included, and overall step length per mouse was averaged from two to three replicates. Zeros were given to any animal with loss of function leading to a complete inability to ambulate.

### Tissue processing and immunohistochemistry

Quadriceps, gastrocnemius, tibialis anterior and triceps muscles, and the liver and epididymal fat pads were excised and weighed upon sacrifice. Transverse paraffin sections of quadriceps and triceps muscles were cut (5 μm) and stained with Masson’s trichrome as previously published [10, 23]. Slides were scanned using an Aperio digital slide scanner. Fat was quantified by manually tracing adipocyte containing regions (previously confirmed by perilipin staining [10]), and data is expressed as a percentage of the total muscle area (fat %). Damage was quantified by manually tracing non-myofiber containing areas (e.g., necrosis and bulk inflammation) [10], and data is expressed as a percentage of total muscle area (damage %). Quantified areas of fat and damage were further subtracted from total muscle area and used to determine the percentage of muscle area occupied by healthy myofibers (healthy %). The percentage of intramuscular collagen deposition or fibrosis was measured using a positive pixel count algorithm in Aperio ImageScope software (hue value of 0.66 and hue width of 0.25) [10]. Collagen-positive pixel area was calculated by multiplying the number of positive pixels by pixel area. Pixel ratios were individually normalized to the average pixel ratio of WT Chow muscles to normalize for detection efficiencies across tissue sections and are thus expressed as a % of WT Chow values (% normalized).

To quantify intramuscular cholesterol levels, transverse frozen sections (5 μm) of quadriceps muscles were also cut and stained with either Filipin (no. SAE0088; SIGMA; 5 mg in 1 ml DMSO) or anti-HMGCR (Invitrogen; no. PA5-37367). Briefly, muscle sections were cut transversely across the mid-belly, mounted onto tragacanth gum (no. G1128; SIGMA) and frozen in liquid nitrogen cooled isopentane. For Filipin, sections were washed in 1X PBS, fixed in 4% PFA in PBS for 15 min, washed in 1X PBS, and then stained for 45 min in working Filipin solution (0.2 ml stock solution in 10 ml PBS). Samples were subsequently washed in 1X PBS, incubated in the nuclei marker DRAQ7 (no. ab109202; Abcam) for 5 min (1:400 in 1× PBS), and then cover slipped using fluorescent mounting media (no. S3023; DAKO). Images were taken on a Zeiss Axio Observer.Z1 microscope and images analyzed using ImageJ software. For HMGCR, sections were washed in 1XPBS, fixed in 4% PFA in PBS for 15 min, and then washed 3 × 5 min in PBS. Sections were blocked for 1 h at RT in 5% normal goat serum (NGS) in PBS, prior to incubation with anti-HMGCR (1:200; Thermo Fisher; no. PA5-37367) overnight at 4 °C. Slides were then washed with 1× PBS and incubated in block solution containing secondary antibody (Thermo Fisher no. A-21245) for 1 h at RT. Following antibody incubation, 3 × 5 min PBS washes were performed, and sections were mounted with VECTASHIELD Mounting Medium with DAPI (Vector Laboratories). Images were taken on a Zeiss AXIO Observer.Z1 microscope and images analyzed using Zeiss imaging software. Quantification was performed by taking the mean fluorescence intensity over 4–5 random images. Sections without incubation in Filipin solution or HMGCR antibodies were used as a negative control and are shown as insets where applicable.

### Immunoblotting

Briefly, frozen gastrocnemius muscles and liver were ground in liquid nitrogen, and the powder was homogenized in ice-cold PBS, 1% NP40, and 1mM EDTA buffer, supplemented with complete EDTA-free protease inhibitor and PhosSTOP phosphatase inhibitor tablets (Roche, Manheim, Germany), and centrifuged at 13,000 g for 20 min at 4 °C and stored at −80 °C until required. Samples were resolved on 10% SDS-PAGE TGX gels (Bio-Rad) and transferred onto nitrocellulose membranes using the Trans Turbo Blot system (Bio-Rad). A total of 30 μg of protein was loaded for liver and skeletal muscle for both LDLR and HMGCR Western blots, respectively. For skeletal muscle concentrations of total and phosphorylated AMPK and rpS6, 50 μg of protein was loaded. Immunoblotting was performed with antibodies to LDLR (Proteintech; no. 66414-1-Ig) and HMGCR (Invitrogen; no. PA5-37367). Antibodies to phosphorylated ribosomal protein S6(Ser235/236; p-rpS6) (no. 4858), total ribosomal protein S6 (no. 2217; t-rpS6), phosphorylated AMP-activated protein kinase α (Thr172) (no. 50081; p-AMPKα) and total AMPKα (no. 5832; t-AMPKα), and the loading control GAPDH (no. 2118S) were from cell signaling. After blocking in Tris-buffered saline with 1% casein (Bio-Rad), primary antibodies incubated in the same medium with the addition of 0.1% Tween20 each diluted 1:1000, except GAPDH (1:2500), and detected using either goat anti-rabbit DyLight680 and anti-mouse DyLight800 (both from Rockland Immunochemicals, no. 611-144-122 and no. 611-145-121; 1:1500-2500) and LI-COR Odyssey scanner. A common sample was loaded onto each gel to normalize for detection efficiencies across membranes.

### Statistical analyses

Statistical analyses are described in the figure legends. Data were analyzed using GraphPad Prism v6. Data are mean ± SEM. Results with *p*-values of less than 0.05 were considered statistically significant.

## Results

### Baseline control and LGMD2B patient characteristics

To assess whether LGMD2B causes lipoprotein abnormalities, biobanked serum samples were selected from LGMD2B and control patients. Population characteristics, age and sex distributions, velocity/speed (m/s) measures for the time to run/walk 10-m test (TTRW), and overall ambulatory status (expressed as a %) are listed in Table 1. As functional TTRW scores were only obtained from ambulant patients, and reduced rates of ambulation were observed in LGMD2B cohorts > 30 years of age, any patient listed as nonambulatory or unable to walk 10 m with usual orthotics and walking aids was assigned a velocity or speed value of “0” and included in the analysis, where a reduced walking speed (m/s) is indicative of poorer ambulatory ability.

**Table 1:** Patient characteristics for serum samples of control and LGMD2B patients

	Control				LGMD2B			
	***Male***	*N*	***Female***	*N*	***Male***	*N*	***Female***	*N*
Age (yrs)								
***Young***	32.4 ± 2.5	*8*	32.3 ± 1.7	*6*	27.3 ± 0.5**	*22*	26.9 ± 0.6*	*20*
***Old***	48.0 ± 2.2####	*7*	45.8 ± 1.6####	*9*	46.3 ± 0.8####	*15*	47.1 ± 0.8####	*18*
TTRW (m/s)								
***Young***	N/A		N/A		1.1 ± 0.2	20	1.4 ± 0.2	20
***Old***					0.6 ± 0.4#	15	0.3 ± 0.2#	13
Ambulatory (%)								
***Young***	N/A		N/A		86%		90%	
***Old***					47%		53%	

Data are mean ± SEM. *m* Meters, *sec* seconds, *%* percentage, *TTRW* time to run/walk 10 m, *yrs* years. The number of patients (*N*) who completed each test/assessment are listed in brackets. Mean values are provided for age and timed tests. Patients listed as nonambulatory were given zeros for TTRW velocity measures. Hash (#) significantly different from young LGMD2B; *P* < 0.0001####. Asterisk (*) significantly different from young control (gender matched); *P* < 0.05*, *P* < 0.01**

Ages of LGMD2B patients with biobanked samples were between 22–30 years (young) and 41–51 years (old), with *N* = 42 and *N* = 33 samples available in each respective group. Biobanked control serum was also sourced from *N* = 14 patients aged between 20 and 39 years (young) and *N* = 16 patients aged between 40 and 55 years (old). The mean ages of patients with available samples were significantly higher in the young control group than the young LGMD2B patients group for both sexes (*P* ≤ 0.05) but was similar among old cohorts (Table 1). Walking/running speeds in old male and female LGMD2B patients were 54% and 81% lower than that of young patients and were associated with an increased percentage of non-ambulation (Table 1).

### LGMD2B patients present with serum HDL-C abnormalities

**Fig. 1:**
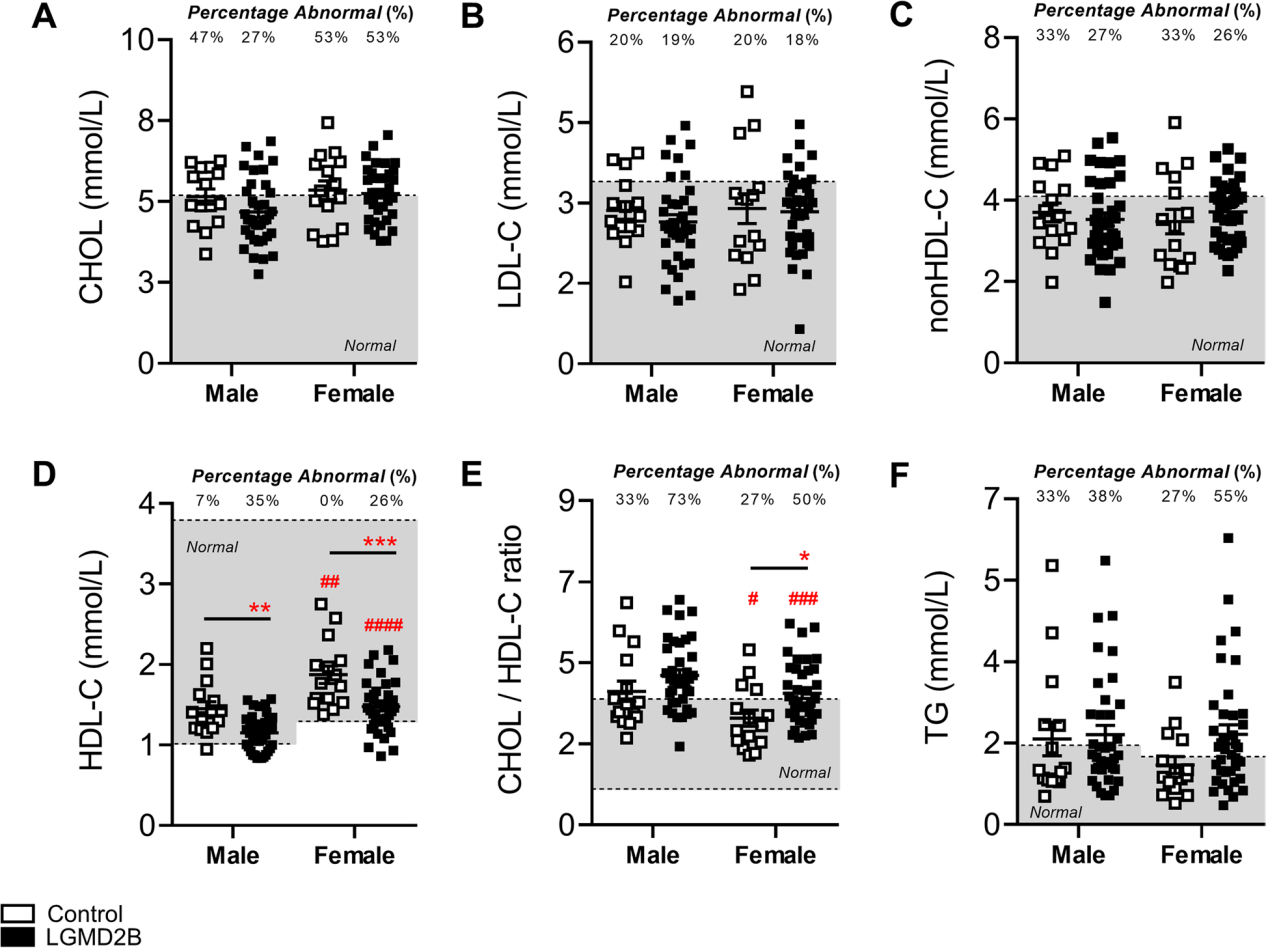
Serum lipoprotein and triglyceride distribution in adult control and LGMD2B patients aged ≥ 20 years and stratified by sex. **A**–**F** Scatter plots of control and LGMD2B lipoprotein serum lipoprotein (CHOL, LDL-C, nonHDL-C, HDL-C, and CHOL/HDL-C ratio) and TGs showing values that fall within and outside of normal adult levels. Two-way ANOVA with Sidak’s post hoc tests were used for direct comparisons between control and LGMD2B means; *P < 0.05; *P < 0.01; ***P < 0.001. Two-way ANOVA with Sidak’s post hoc tests were used for direct comparisons between male and female means; #*P* < 0.05; ##*P* < 0.01; ###*P* < 0.001. Gray zone denotes normal adult range for each specific parameter. The number of patients falling outside of normal range (abnormal values) is listed as a percentage. Mean ± SEM. Male control (*N* = 15). female control (*N* = 15). Male LGMD2B (*N* = 37). Female LGMD2B (*N* = 38)

**Fig. 2:**
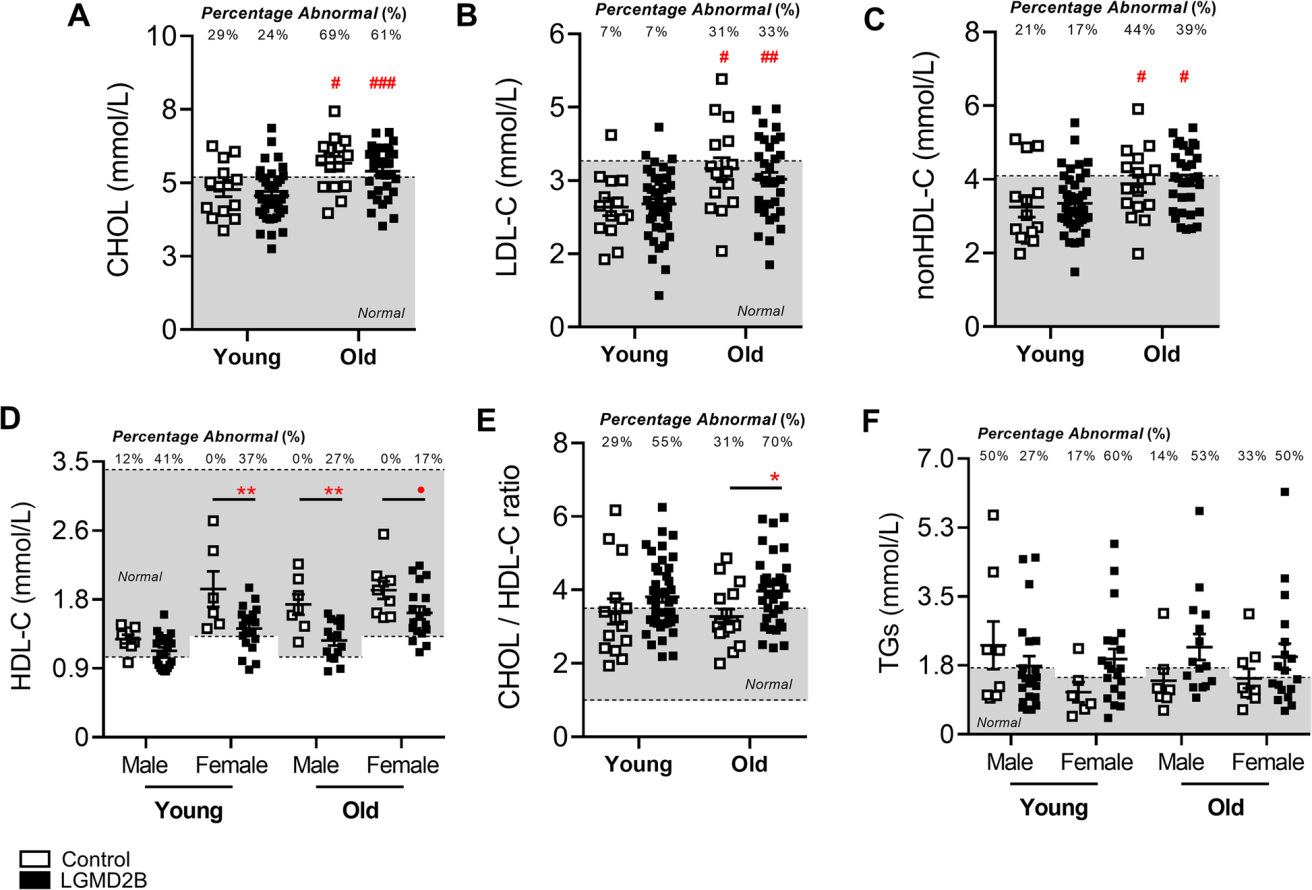
Serum lipoprotein and triglyceride distribution in adult control and LGMD2B patients aged ≥ 20 years and stratified by age. **A**–**F** Scatter plots of control and LGMD2B lipoprotein serum lipoprotein (CHOL, LDL-C, nonHDL-C, HDL-C, and CHOL/HDL-C ratio) and TGs showing values that fall within and outside of normal adult levels. **A**, **B**, **C**, and **E** Two-way ANOVA with Sidak’s post hoc tests were used for direct comparisons between control and LGMD2B means; *P < 0.05; **P < 0.01; ***P < 0.001. **D** and **F** Two-way ANOVA with Sidak’s post hoc tests were used for direct comparisons between control and LGMD2B means; #*P* < 0.05; ##*P* < 0.01; ###*P* < 0.001. Gray zone denotes normal adult range for each specific parameter. The number of patients falling outside of normal range (abnormal values) is listed as a percentage. Mean ± SEM. **A**, **B**, **C**, and **E** Young control (*N* = 14); old control (*N* = 16); young LGMD2B (*N* = 42); old LGMD2B (*N* = 33). **D** and **F** Young male control (*N* = 8); young female control (*N* = 6); old male control (*N* = 7); old female control (*N* = 9); young male LGMD2B (*N* = 22); young female LGMD2B (*N* = 20); old male LGMD2B (*N* = 15); old female LGMD2B (*N* = 18)

When serum lipoprotein levels were stratified by sex, both male and female LGMD2B patients displayed statistically similar levels of CHOL, LDL-C, and nonHDL-C compared to their respective controls (Fig. 1A–C). Interestingly, we observed a 21% and 19% reduction in LGMD2B serum HDL-C levels in both male and female LGMD2B patients, respectively (Fig. 1D; *P* < 0.01). Analysis of CHOL/HDL-C ratios, a typical indicator of cardiovascular disease, demonstrated that 73% of male and 50% of female LGMD2B patients had abnormal CHOL/HDL-C ratios (Fig. 1E). CHOL/HDL-C ratios were also 12% higher in males (*ns*) and 25% higher in females than their respective controls (*P* < 0.05; Fig. 1E). TG levels in LGMD2B patients of both sexes were not statistically different from controls (Fig. 1F). When stratified for age (young vs old), LGMD2B patients displayed similar levels of serum CHOL, LDL-C, and nonHDL-C compared to age-matched control samples (Fig. 2A–C). Significant decreases in mean serum HDL-C, however, were observed in young LGMD2B females (by 27%), as well as in older LGMD2B patients regardless of sex (27% male and 15% female, respectively; Fig. 2D). Similarly, CHOL/HDL-C ratios were significantly increased in old (21%; *P* < 0.05) LGMD2B patients compared to controls, yet both young and old LGMD2B patients showed an unusually high prevalence of ratio abnormalities (55% young and 70% old) (Fig. 2E). TG levels were similar across all cohorts (Fig. 2F).

### LGMD2B causes subclinical liver enzyme elevations

**Fig. 3:**
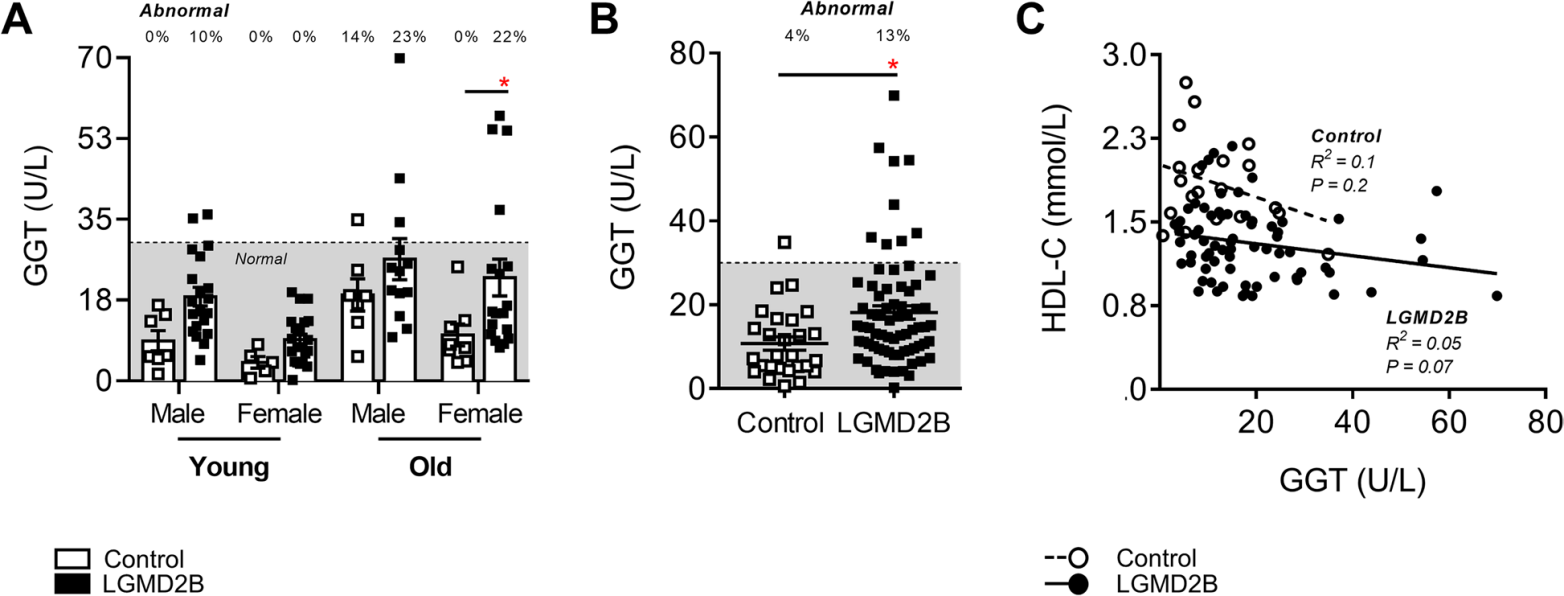
Comparative analysis of male and female serum gamma-glutamyl transferase (GGT) levels and overall distribution in adult control and LGMD2B patients ≥ 20 years of age. **A** Scatter plot of control and LGMD2B serum GGT stratified for both age and sex. Two-way ANOVA with Sidak’s post hoc tests were used for direct comparisons between control and LGMD2B means; *P < 0.05. **B** Scatter plot of combined control and LGMD2B serum GGT. Unpaired *t*-test was used to compare means; *P < 0.05. Gray zone denotes normal clinical adult range. **C** Correlations between serum GGT and HDL-C levels in control and LGMD2B patients. Pearson correlation *R*^2^ and *P*-values are listed were applicable. Mean ± SEM. **A** Young male control (*N* = 7); young female control (*N* = 5); old male control (*N* = 7); old female control (*N* = 8); young male LGMD2B (*N* = 20); young female LGMD2B (*N* = 19); old male LGMD2B (*N* = 13); old female LGMD2B (*N* = 18). **B**–**C** Control (*N* = 27); LGMD2B (*N* = 70)

To eliminate liver damage as the source of low-serum HDL-C levels in LGMD2B patients, analyses of serum gamma-glutamyl transferase (GGT), the most sensitive enzymatic indicator of liver disease, were performed in the abovementioned samples (Fig. 3). In the context of MD, regular markers of liver abnormality including alanine and aspartate transaminases (ALT and AST) are typically deemed inappropriate, as transaminases are also released by muscle cells in response to damage [26]. When stratified for both age and sex, GGT levels were increased in young male (by 110%; *ns*), young female (by 122%; *ns*), old male (by 44%; *ns*), and old female (123%; *P* < 0.05) LGMD2B cohorts compared to healthy controls (Fig. 3A). Overall, GGT levels in LGMD2B patients were 59% higher than that of healthy controls (*P* < 0.05), although only 13% of all LGMD2B patients exhibited values that were considered to be clinically abnormal (Fig. 3B). Moreover, no significant correlation between serum GGT and HDL-C levels was observed (Fig. 3C).

### Simvastatin did not attenuate murine LGMD2B-associated disease pathology despite showing biological activity

**Fig. 4:**
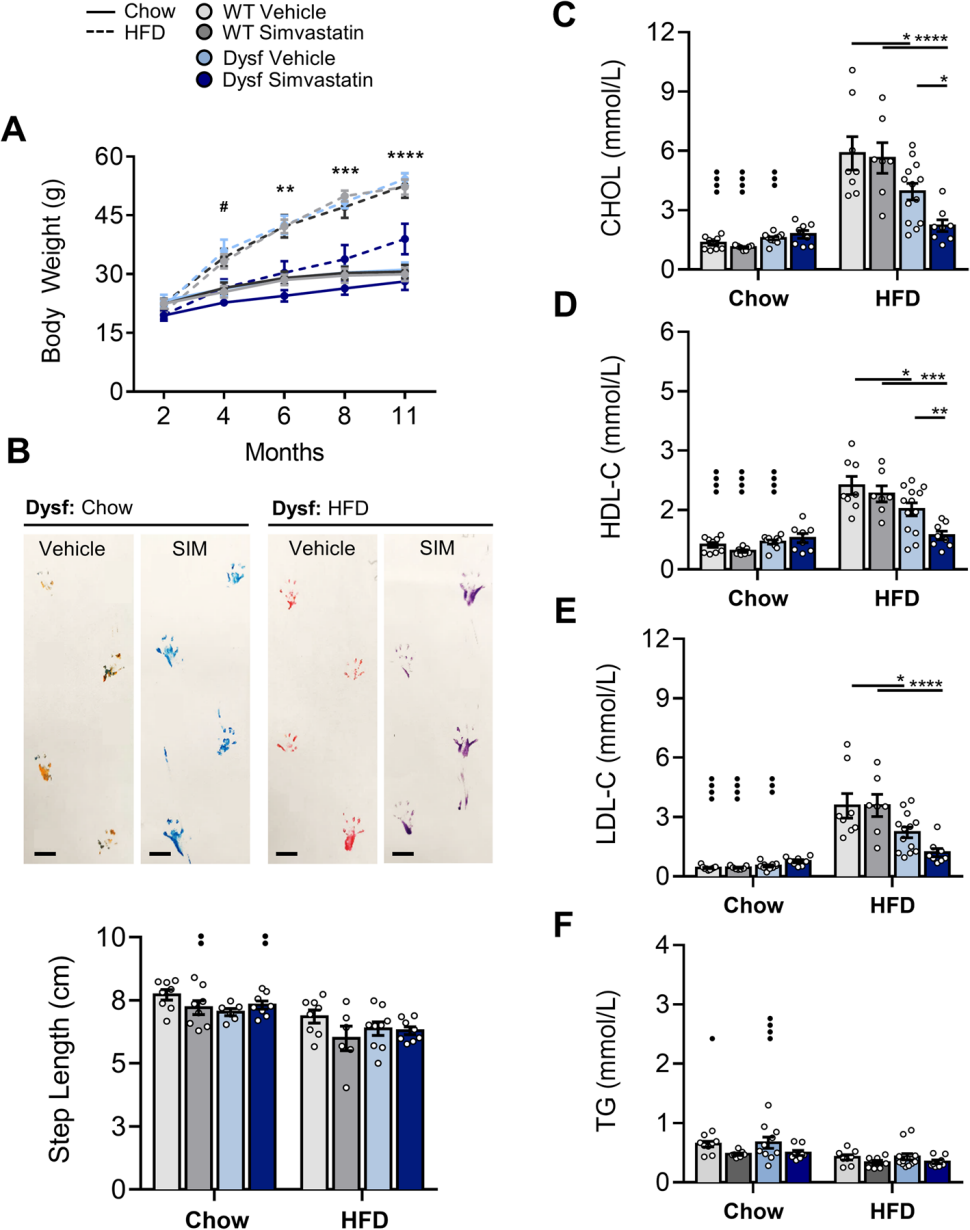
Simvastatin mitigates HFD-induced weight gain and plasma lipoprotein accretion in Dysf-null mice but fails to prevent ambulatory dysfunction. **A** Monthly body weight measures (2, 4, 6, 8, and 11 months) in Chow and HFD-fed, vehicle, and simvastatin (SIM)-treated WT and Dysf mice. Two-way ANOVA with Tukey’s post hoc tests were used for direct mean comparisons; #*P* < 0.05 and compares WT SIM and Dysf SIM at the age group selected. **P < 0.01 and ***P < 0.001 independently compare both WT SIM to Dysf SIM and Dysf vehicle to Dysf SIM across the age groups selected. **B** End-point (11 m) step length images from Chow and HFD-fed Dysf mice treated with either vehicle or simvastatin and their quantification for all groups. Two-way ANOVA with Sidak’s post hoc tests were used for direct mean comparisons between; ••*P* < 0.01 and within; no significance detected. **C**–**E** End-point total plasma cholesterol (CHOL), high-density lipoprotein cholesterol (HDL-C), low-density lipoprotein cholesterol (LDL-C) and triglycerides (TG) from Chow and HFD-fed, WT and Dysf-null mice treated with either vehicle or simvastatin. Two-way ANOVA with Sidak’s post hoc tests were used for direct mean comparisons between, •••*P* < 0.001 and ••••*P* < 0.0001, and within, *P < 0.05 and ****P < 0.0001. Mean ± SEM. **A**–**B***N* = 6–9. **C**–**F***N* = 8–13

Since statin inhibition of muscle wasting in dystrophin-deficient *mdx* models of DMD remains controversial [15, 16], we tested simvastatin in the Dysf-null model of LGMD2B between 2 and 11 months, under both normolipidemic chow and hyperlipidemic HFD conditions. In Dysf mice, oral administration of simvastatin prevented HFD-induced weight gain (Fig. 4A), owing to both a reduction in epidydimal fat accumulation and liver enlargement (Supp. Fig. 2), yet simvastatin had no effect on ambulatory function and step length readouts (Fig. 4B). Notably, simvastatin did not change CHOL, HDL-C, or LDL-C levels in chow-fed mice and had a minor effect on TGs (Fig. 4C–F). In HFD-fed animals, Dysf mice showed lower levels of CHOL, HDL-C, LDL-C, and TGs than their WT controls (Fig. 4C–F), and simvastatin induced a 46% and 43% reduction in plasma CHOL and HDL-C but left TG levels unaffected (Fig. 4C–E). Direct comparisons between vehicle treated WT and Dysf-null lipid levels have been included in Supp. Fig. 2A–F. No discernable effects of simvastatin were observed on body, fat pad, or liver weights in chow-fed mice or WT controls on either diet (Supp. Fig. 3A–B).

**Fig. 5:**
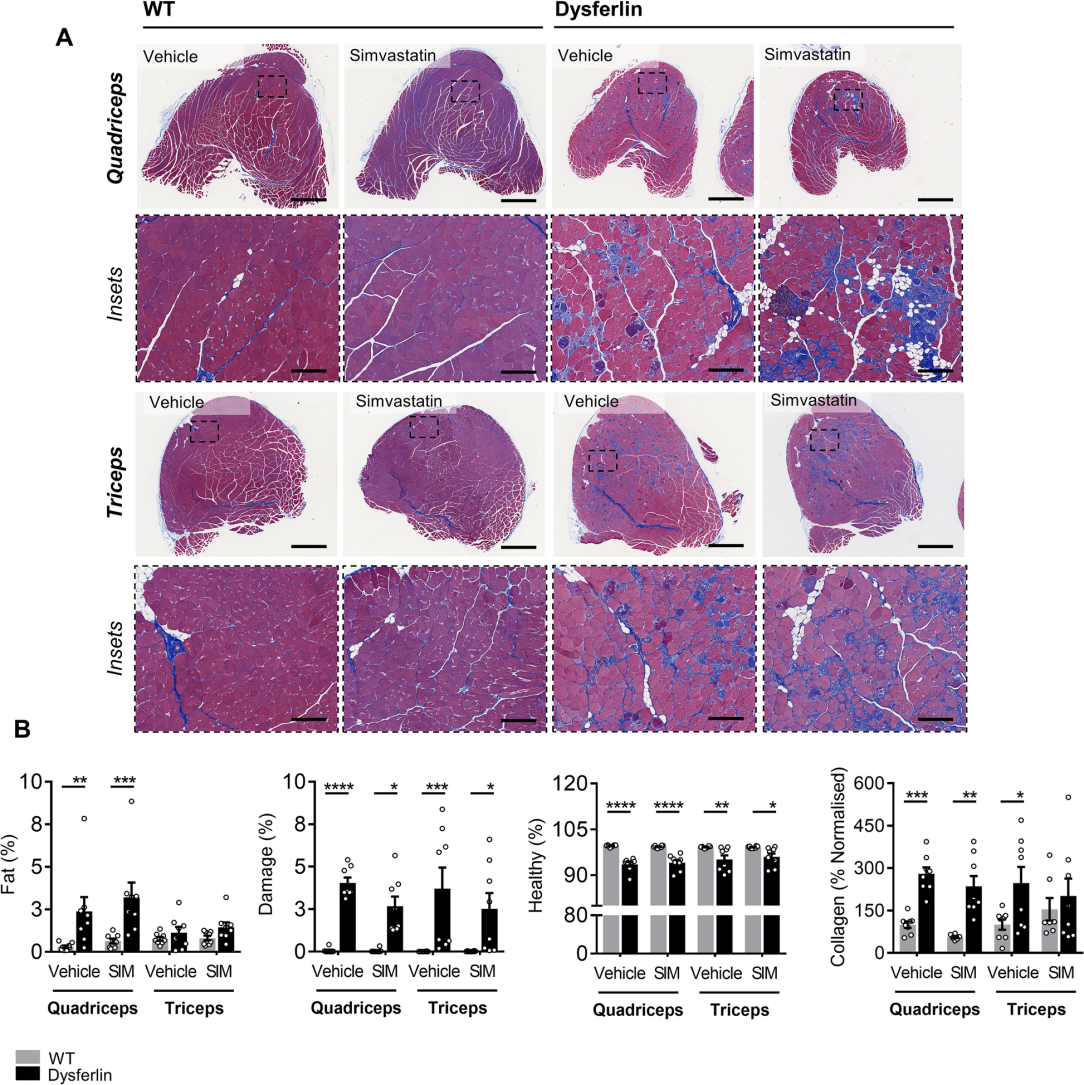
Simvastatin did not attenuate LGMD2B-associated quadriceps or triceps histopathology in normolipidemic Chow-fed Dysf-null mice. **A** Representative images of and **B** quantification of percentage fat, damaged and healthy myofiber areas, and the percentage of collaged deposition in whole quadriceps and tricep muscles from Chow-fed WT and Dysf mice treated with either vehicle or SIM. Scale 1 mm for whole muscle images and 200 μm for insets. Two-way ANOVA with Sidak’s post hoc tests were used for direct comparisons between WT and Dysf mice; *P < 0.05; **P < 0.01; ***P < 0.001; ****P < 0.0001. Mean ± SEM. **A**–**B***N* = 7–8

**Fig. 6:**
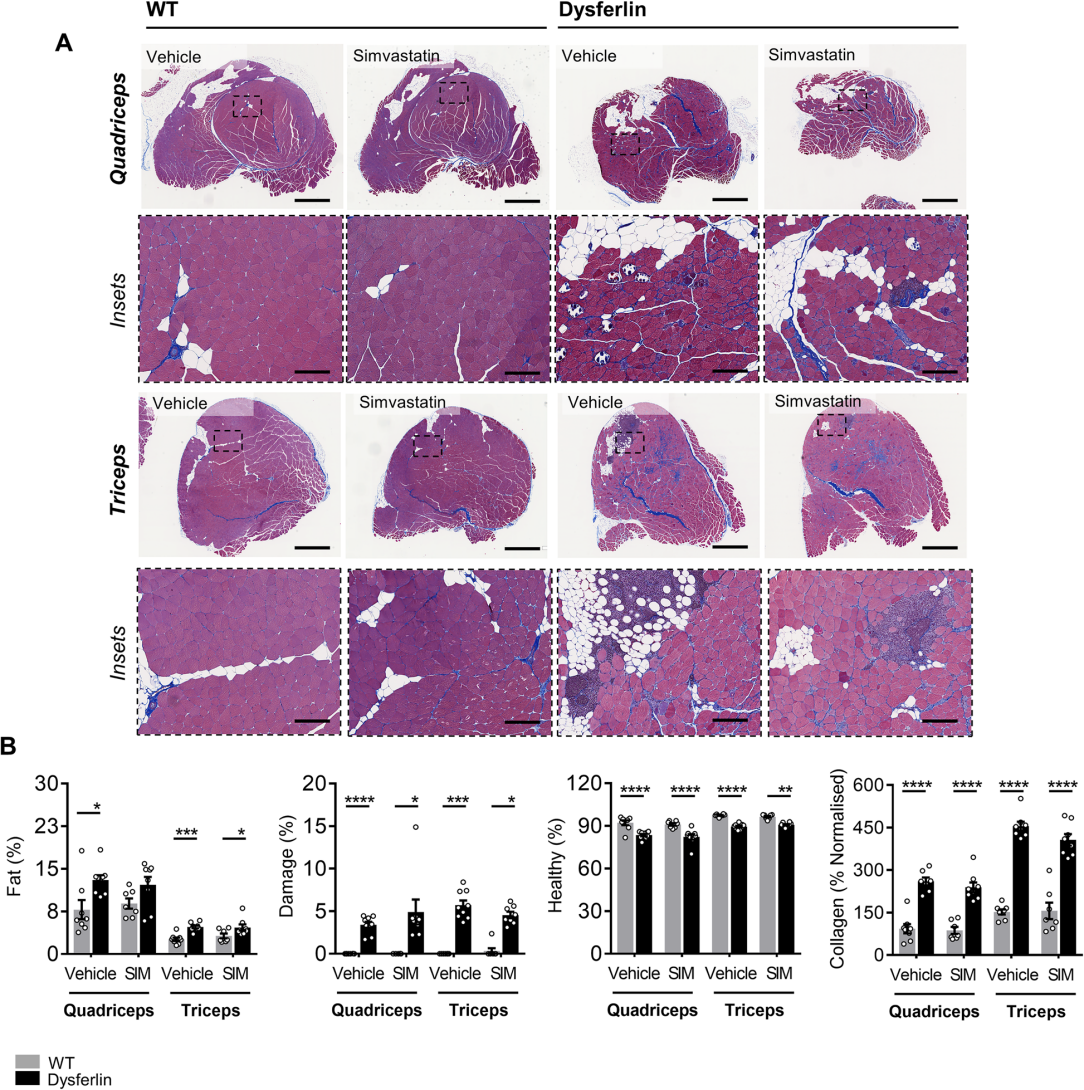
Simvastatin did not attenuate LGMD2B-associated quadriceps or triceps histopathology in hyperlipidemic HFD-fed Dysf-null mice. **A** Representative images of and **B** quantification of percentage fat, damaged and healthy myofiber areas, and the percentage of collaged deposition in whole quadriceps and tricep muscles from HFD-fed WT and Dysf mice treated with either vehicle or SIM. Scale 1 mm for whole muscle images and 200 μm for insets. Two-way ANOVA with Sidak’s post hoc tests were used for direct comparisons between WT and Dysf mice; *P < 0.05; **P < 0.01; ***P < 0.001; ****P < 0.0001. Mean ± SEM. **A**–**B***N* = 7–9

Despite profoundly affecting body composition and key metabolic parameters, which confirmed biological activity, simvastatin failed to prevent LGMD2B-associated muscle wasting in quadriceps and triceps muscles under either dietary condition (Table 2). Moreover, in quadriceps and triceps muscle sections stained with Masson’s trichrome, simvastatin was unable to mitigate LGMD2B-associated fat infiltration, muscle damage, and collagen deposition in chow and HFD-fed Dysf mice (Figs. 5A–B and 6A–B). No detrimental effects of simvastatin on muscle pathology were observed in age- and diet-matched WT mice (Figs. 5A–B and 6A–B).

**Table 2:** Effect of HFD-feeding and simvastatin treatment on relative muscle weights in C57BL/6 and Dysf-null mice aged 11 months

	Vehicle		Simvastatin	
	*WT*	*Dysf*	*WT*	*Dysf*
**Chow**				
*Quadriceps*	12.4 ± 0.4	10.6 ± 0.4	12.4 ± 0.7	10.2 ± 0.8
*Gastrocnemius*	8.7 ± 0.4	9.4 ± 0.6	8.7 ± 0.4	8.2 ± 0.7
*Tibialis anterior*	3.1 ± 0.2	3.0 ± 0.2	2.9 ± 0.1	2.8 ± 0.1
*Triceps brachii*	6.6 ± 0.3	7.1 ± 0.3	6.6 ± 0.5	6.4 ± 0.4
**HFD**				
*Quad*	12.6 ± 0.3	9.3 ± 0.5*	12.6 ± 0.6	9.0 ± 0.8*
*Gastrocnemius*	9.0 ± 0.2	8.2 ± 0.4*	9.6 ± 0.3	7.5 ± 0.4*
*Tibialis anterior*	2.7 ± 0.1	2.8 ± 0.1	3.0 ± 0.2	2.9 ± 0.1
*Triceps brachii*	6.7 ± 0.3	6.3 ± 0.6	6.9 ± 0.3	5.9 ± 0.3

Muscle weights (mg) were weighed and quantified relative to tibia length (mm). Data are mean ± SEM. Asterisk (*) indicates significantly different compared to C57BL/6 (WT) cohorts for each listed parameter (*) *P* < 0.5; two-way ANOVA with Tukey’s post hoc tests. No significant differences between Chow and HFD cohorts were detected; two-way ANOVA with Sidak’s post hoc tests

### Dysf deficiency causes overexpression of cholesterol metabolism regulators in skeletal muscle but not the liver

**Fig. 7:**
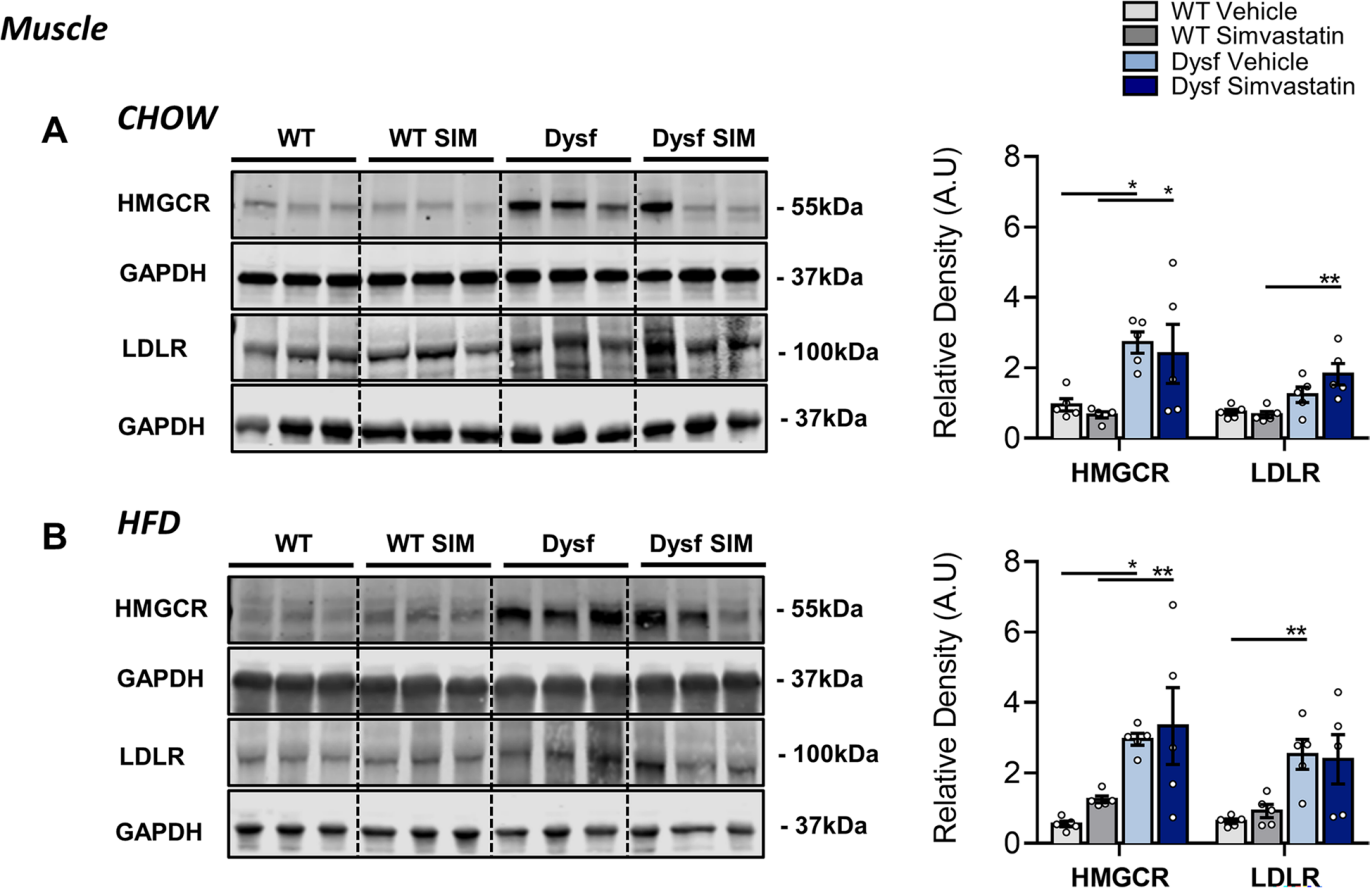
Simvastatin failed to downregulate HMGCR and LDLR signaling in skeletal muscles in Chow or HFD-fed Dysf-null mice. **A** Representative images and immunoblot quantification of HMGCR and LDLR in gastrocnemius muscles of Chow-fed, WT and Dysf mice treated with either vehicle or simvastatin. Two-way ANOVA with Sidak’s post hoc tests were used for direct mean comparisons; *P < 0.05, **P < 0.01, and ****P < 0.0001. **B** Representative images and immunoblot quantification of HMGCR and LDLR in gastrocnemius muscles of Chow-fed, WT and Dysf mice treated with either vehicle or simvastatin. Two-way ANOVA with Sidak’s post hoc tests were used for direct mean comparisons; *P < 0.05, **P < 0.01, and ****P < 0.0001. HMGCR and LDLR were standardized to the loading control GAPDH. *Y*-axes represent arbitrary units (AU). Full blots were imaged separately and thus have differing exposures. A common sample was loading onto each gel to normalize for detection efficiencies across membranes. Mean ± SEM. *N* = 5 for each cohorts

**Fig. 8:**
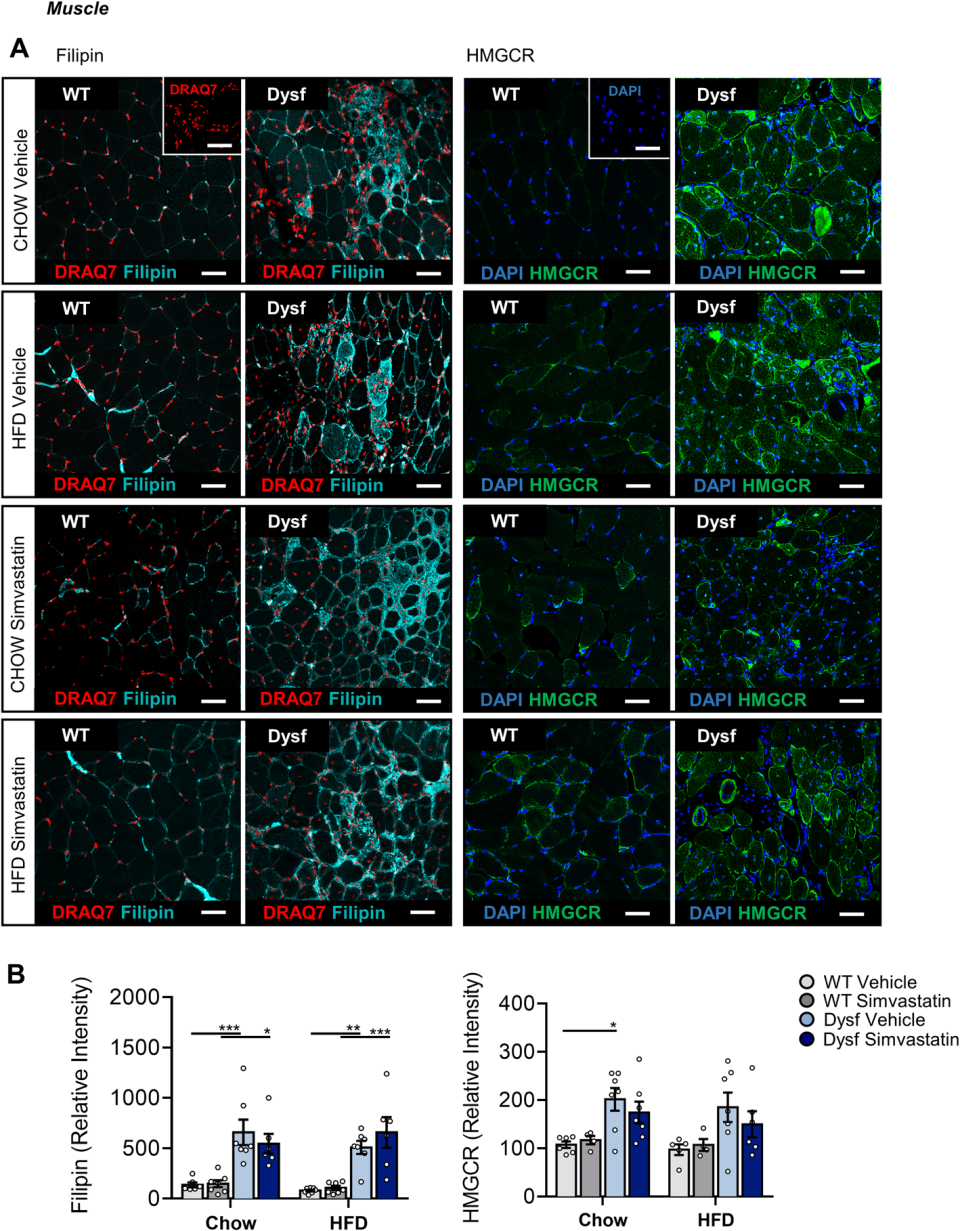
Simvastatin failed to normalize intramuscular levels of Filipin and HMGCR in the quadriceps of Chow or HFD-fed Dysf-null mice. **A** Representative images of Filipin and HMGCR in both Chow and HFD-fed, WT and Dysf mice treated with either vehicle or simvastatin. **B** Quantification of the average fluorescent intensity of Filipin and HMGCR. Relative intensity values were generated by normalizing to WT (Chow) levels to account for detection efficiencies. Scale 50 μm in both main images and insets. Insets: no Filipin control (DRAQ7 only) and no HMGCR control DAPI only). Two-way ANOVA with Sidak’s post hoc tests were used for direct comparisons between WT and Dysf mice; *P < 0.05; **P < 0.01; ***P < 0.001. Mean ± SEM. N = 4–7 per cohort

**Fig. 9:**
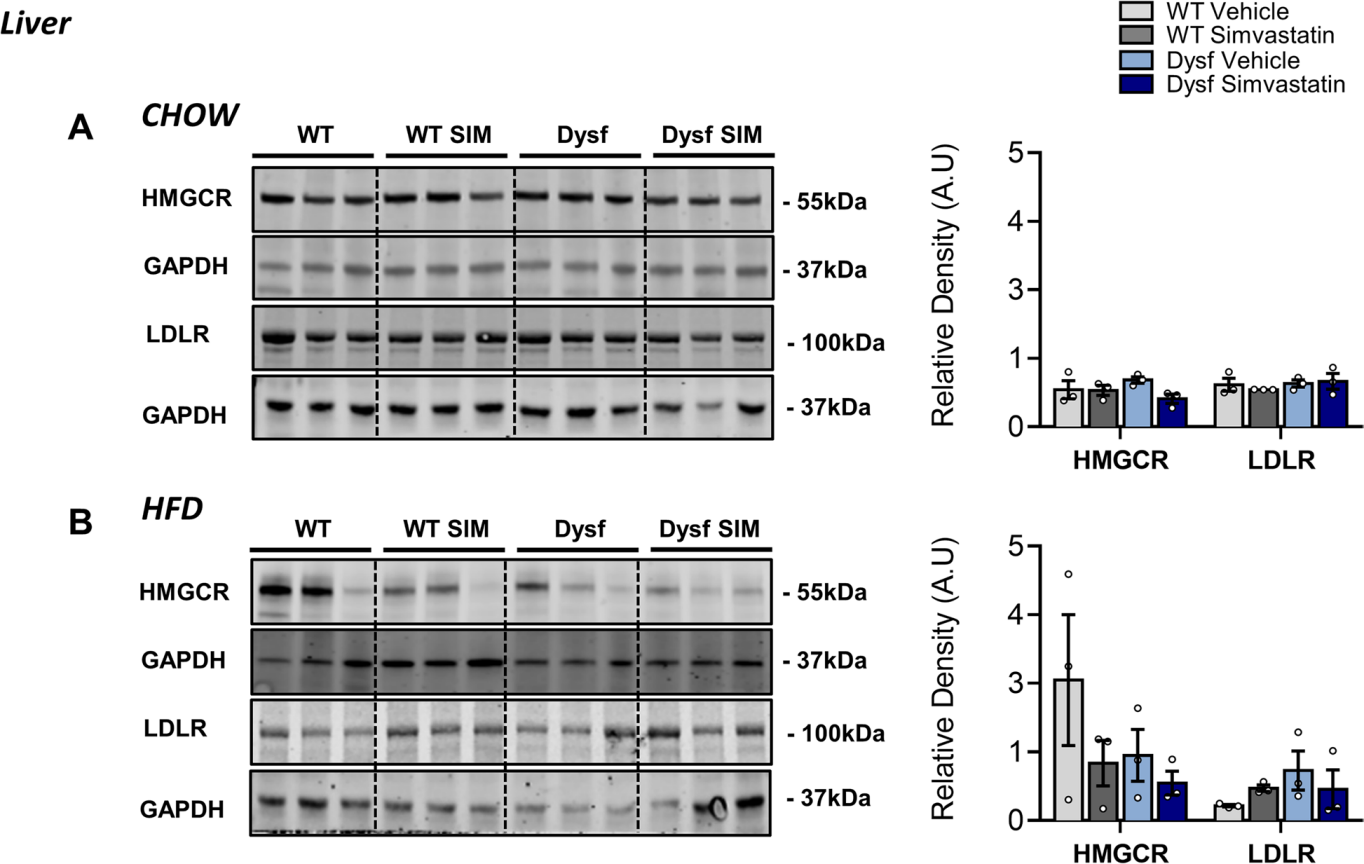
No effect of simvastatin on the liver expression of HMGCR or LDLR. **A** Representative images and immunoblot quantification of HMGCR and LDLR in the livers of Chow-fed, WT and Dysf mice treated with either vehicle or simvastatin. Two-way ANOVA with Sidak’s post hoc tests were used for direct mean comparisons; *no significance*. **B** Representative images and immunoblot quantification of HMGCR and LDLR in the livers of Chow-fed, WT, and Dysf mice treated with either vehicle or simvastatin. Two-way ANOVA with Sidak’s post hoc tests were used for direct mean comparisons; no significance. HMGCR and LDLR were standardized to the loading control GAPDH. *Y*-axes represent arbitrary units (AU). Full blots were imaged separately and thus have differing exposures. A common sample was loading onto each gel to normalize for detection efficiencies across membranes. Mean ± SEM. *N* = 3 for each cohort

To further investigate the role of cholesterol in LGMD2B muscle pathology, we measured muscle protein levels of HMGCR and LDLR, the main regulators of nonHDL-C cholesterol metabolism and effectors of statin bioactivity. Western blot analysis demonstrated that HMGCR and LDLR protein expression was increased in gastrocnemius muscles of chow and HFD-fed Dysf mice compared to diet-matched WT controls, and simvastatin treatment failed to normalize intramuscular levels of either protein (Fig. 7B). These data were further corroborated using immunofluorescence tissue staining for both HMGCR and Filipin, the latter of which detects cholesterol-rich domains as well as intracellular levels of unesterified-free cholesterol [15]. Filipin staining was significantly elevated in Dysf-null quadriceps muscle sections compared to WT under both chow (by 380%; *P* < 0.001) and HFD-fed conditions (by 775%; *P* < 0.001) (Fig. 8A–B). Similarly, levels of HMGCR were elevated by 87% (*P* < 0.05) and 94% (*ns*) in the quadriceps muscles of Chow and HFD-fed Dysf-null mice compared to WT controls, respectively (Fig. 8A–B). Intramuscular levels of either protein were again not significantly reduced following simvastatin treatment (Fig. 8A–B). In the liver, the main site of whole-body cholesterol regulation, we observed that in contrast to skeletal muscle, neither HMGCR nor LDLR were affected by genotype, diet, or simvastatin treatment (Fig. 9A–B). Combined, these data suggest that loss of dysferlin interferes with normal muscle expression of mevalonate/HMGCR and LDLR, two key regulators of cholesterol metabolism previously shown to be both elevated in *mdx* muscle tissues and reduced with simvastatin [15].

### Simvastatin treatment had no effect on mTORC1 or AMPK pathway activation in muscle lysates

mTORC1 signalling is a key regulator of protein synthesis in skeletal muscle. The activation of mTORC1, either downstream of AKT or directly by nutrients, can promote protein synthesis by phosphorylating two major targets: S6K1 and 4E-BP1, which can also be assessed downstream by rpS6(Ser235/236) expression [27]. Since simvastatin has been shown to inhibit the phosphorylation of rpS6 (Ser235/236; p-rpS6) and mTORC1 signalling in skeletal muscle and contribute to statin-induced myopathy [28], we therefore measured rates of p-rpS6 activation in skeletal muscle lysates of WT and Dysf-null mice treated with simvastatin. Western blot analysis demonstrated that simvastatin had no effect on levels of p-rpS6(Ser235/236) levels when standardized to total rpS6 (t-rpS6) (Supp. Fig. 4A and B). Levels of total rpS6 levels (t-rpS6) standardized to GAPDH in Dysf-null tissues were also unaffected by simvastatin treatment, despite displaying increased t-rpS6 protein standardized to GAPDH when compared to WT muscle (Supp. Fig. 4A and B). While AMPK phosphorylation is an inhibitory regulator of the mTORC1 pathway [29], in other tissues (e.g., liver), the phosphorylation of AMPKα(Thr172) can also directly downregulate HMGCR activity and thus inhibit intracellular rates of cholesterol synthesis [30], although it should be noted that studies describing AMPK-mediated inhibition of cholesterol synthesis are limited. Similarly to mTORC1 activation, levels of p-AMPKα(Thr172) standardized to t-AMPKα were not significantly affected by simvastatin treatment in WT and Dysf-null mice fed either a chow or HFD (Supp. Fig. 4A and C). While total amounts of AMPKα standardized to the loading control GAPDH were significantly elevated in both chow and HFD-fed Dysf-null muscles compared to WT (by 113% and 200%, respectively), expression levels were not sufficient to antagonize intramuscular cholesterol synthesis (nor affect mTORC1 basal activation) (Supp. Fig. 4A and C). Furthermore, levels of t-AMPKα were not affected by simvastatin treatment (Supp. Fig. 4A and C).

## Discussion

The current study is the first to comparatively analyze serum lipid profiles from both LGMD2B and control individuals using routine clinical approaches and report that LGMD2B patients display significant reductions in protective HDL-C, regardless of age or sex differences. Importantly, the comparative assessment of plasma lipid profiles isolated from mice studied both herein and as previously reported confirmed Dysf-associated lipoprotein abnormalities at a late disease stage [31], providing evidence of a major metabolic component to LGMD2B. The absence of clinically elevated GGT in LGMD2B samples argues against liver damage as its primary cause, and while transaminases have been reported to be elevated in LGMD2B, it is typically in response to muscle rather than liver damage [26]. However, despite significant genetic heterogeneity between types of MD, we have also described clinically significant lipoprotein abnormalities in human and canine DMD, which together with the current study support the novel theory that MDs represent a new class of genetic dyslipidemias. Indeed, hypercholesterolemia and elevated TG have also been reported in LGMD type 1C [32] (caveolin-3 mutations) and myotonic dystrophies types 1 and 2 (DMPK and CNBP mutations, respectively) [33, 34] — although often in the absence of control conditions or using experimental approaches.

Our analyses paired with that of recent studies suggest that MD correlates with perturbed lipid metabolism regulators, although whether this occurs in primary or secondary fashion to muscle wasting is unknown. Recent miRNA profiling of DMD samples has highlighted the dysregulation of sterol-regulatory binding proteins (SREBPs) and downstream mevalonate pathway mediators, and these findings are further supported by our current analyzes of HMGCR, LDLR, and Filipin in LGMD2B muscle. Of note, patients that display elevated anti-HMGCR antibodies exhibit prominent LGMD-like myopathy [35]. Increased deposition of ApoB-containing lipoproteins in muscle, such as LDL-C, can also increase inflammatory cell recruitment, which was a key observation in our dyslipidemic models of DMD and LGMD2B [11, 23], and potentially rationalizes the misdiagnosis of LGMD2B for polymyositis. As HDL-C plays a critical role in reverse cholesterol transport (RCT) and exerts its protective effects by shuttling excess cholesterol from peripheral tissues to the liver for biliary disposal [36], an HDL-C deficiency may lead to deleterious muscle cholesterol accumulation, although HDL-C can also mediate intracellular glucose and mitochondrial homeostatic processes in skeletal muscle independently of its role in lipid transport [37]. Such lipometabolic abnormalities may therefore act to synergistically affect intracellular cholesterol mechanisms, as the cholesterol content of myofibers appears to be tightly regulated. Indeed, skeletal muscle-specific deletion of HMGCR induces severe and early skeletal muscle atrophy [38], despite muscle having considerably little de novo cholesterol synthesis [39]. The accumulation of multiple lipid species, including cholesterol, sphingolipids, phospholipids, and the overexpression of the genes that regulate their metabolism, however has been extensively reported in dysferlin-deficient rodent [40] and LGMD2B patient muscle biopsies [41].

The possibility of treating MD with lipid-lowering medications is an interesting concept that deserves greater consideration. In severe *mdx* and Dysf mice lacking ApoE, we showed that the cholesterol absorption blocker ezetimibe can be targeted to attenuate disease severity [42]. In mild *mdx* mice, simvastatin can block intramuscular inflammation and fibrosis [15] and improve functional skeletal muscle, diaphragm, and cardiac parameters via anti-inflammatory and anti-oxidative pleiotropism [15–17], although others have failed to replicate these therapeutic properties with either simvastatin [19, 20] or rosuvastatin [18]. In our study, we demonstrate that simvastatin does not reduce CHOL levels in chow fed Dysf mice yet does reduce HDL-C levels in HFD groups as well as muscle-specific HMGCR and LDLR protein expression which may rationalize its lack of therapeutic properties in our model of LGMD2B. As mice have very different cholesterol and lipoprotein metabolism than humans, whether this is due to an absence of therapeutic pleiotropism or abnormal cholesterol-dependent responses is unknown; humans carry most of their serum cholesterol in LDL-C, whereas mice are typically rich in HDL-C [43]. In patients, statins mainly reduce LDL-C and have mild HDL-C potentiating effects, in stark contrast to what we report herein. It is likely, however, that Dysf likely affects HMGCR and LDLR homeostasis, which may result in an abnormal response to statin therapy. Whether statins remain a viable treatment option in MD patients is debatable in light of reported risk factors, including myopathy, myositis, and rhabdomyolysis (reviewed in [44]). Yet, we observed no further deleterious effects of simvastatin in the muscles of either WT or Dysf mice, which accords with principle studies using the same or similar dosages and route of administration [16]. It is possible that patients with differing forms of MD could respond heterogeneously to the modulation of either lipoprotein metabolism (LDL-C) or intracellular cholesterol synthesis (HMGCR), with potentially unexpected compensatory responses between these two pathways, since statins induce major LDLR upregulation.

## Supplementary Information

**Additional file 1: Supp. Figure 1.** Correlations between serum HDL-C and functional TTRW scores in LGMD2B patients ≥20 years of age. A: Correlative scatter plots stratified for sex, and B: age. Pearson correlation *R*^2^ and *P* values are listed were applicable. A: LGMD2B Male *N* = 35, LGMD2B Female *N* = 32; B: young LGMD2B *N* = 39; old LGMD2B *N* = 28.

**Additional file 2: Supp. Figure 2.** Plasma lipoprotein and triglyceride distribution in Chow and HFD-fed WT and Dysf-null mice, aged 11m. A-F: scatter plots of WT and Dysf-null plasma lipoprotein (CHOL, LDL-C, nonHDL-C, HDL-C and CHOL/HDL-C ratio) and TGs. Two way ANOVA with Sidak’s post hoc tests were used for direct comparisons between WT and Dysf-null means; (*) *P* < 0.05. Two way ANOVA with Sidak’s post hoc tests were used for direct comparisons between Chow and HFD means; (#) *P* < 0.05; (##) *P* < 0.01; (###) *P* < 0.001. Mean±SEM. A-F: *N* = 8-12.

**Additional file 3: Supp. Figure 3.** Simvastatin mitigates HFD-induced abdominal adiposity and liver enlargement in Dysf-null mice. A: End-point epidydimal fat pad and B: liver weights in in Chow and HFD-fed, vehicle and simvastatin (SIM) treated, WT and Dysf mice (11mo). Two way ANOVA with Sidak’s post hoc tests were used for direct mean comparisons between; (••••) *P* < 0.0001, and within; (**) *P* < 0.01; and (***) *P* < 0.001. Mean±SEM. *N* = 7-9 for each cohort.

**Additional file 4: Supp. Figure 4.** Simvastatin treatment had no effect on mTORC1 or AMPK pathway activation in Dysf-null muscle lysates: A: Representative immunoblot images for p-rpS6(Ser235/236), t-rpS6, p-AMPKα(Thr172), t-AMPKα and GAPDH in quadriceps muscles of Chow-fed, WT and Dysf mice treated with either Vehicle or Simvastatin. B: Immunoblot quantification of p-rpS6(Ser235/236) / t-rpS6 and t-rpS6 / GAPDH. Two way ANOVA with Sidak’s post hoc tests were used for direct mean comparisons; (*) *P* < 0.05; (**) *P* < 0.01; and (****) *P* < 0.0001. B: Immunoblot quantification of p-AMPKα(Thr172), / t-AMPKα and t-AMPKα / GAPDH. Two way ANOVA with Sidak’s post hoc tests were used for direct mean comparisons; (**) *P* < 0.01; (***) *P* < 0.001; and (****) *P* < 0.0001. Y-axes represent arbitrary units (A.U). Full blots were imaged separately and thus have differing exposures. A common sample was loading onto each gel to normalize for detection efficiencies across membranes. Mean±SEM. *N* = 4 for each cohort.

## Data Availability

Data will be made available upon reasonable request.
