# Supplementary figures and images for: Limb-girdle muscular dystrophy type 2B causes HDL-C abnormalities in patients and statin-resistant muscle wasting in dysferlin-deficient mice
Source: Skelet Muscle. 2022 Nov 29;12:25. doi: 10.1186/s13395-022-00308-6 (PMC9706908; doi:10.1186/s13395-022-00308-6)

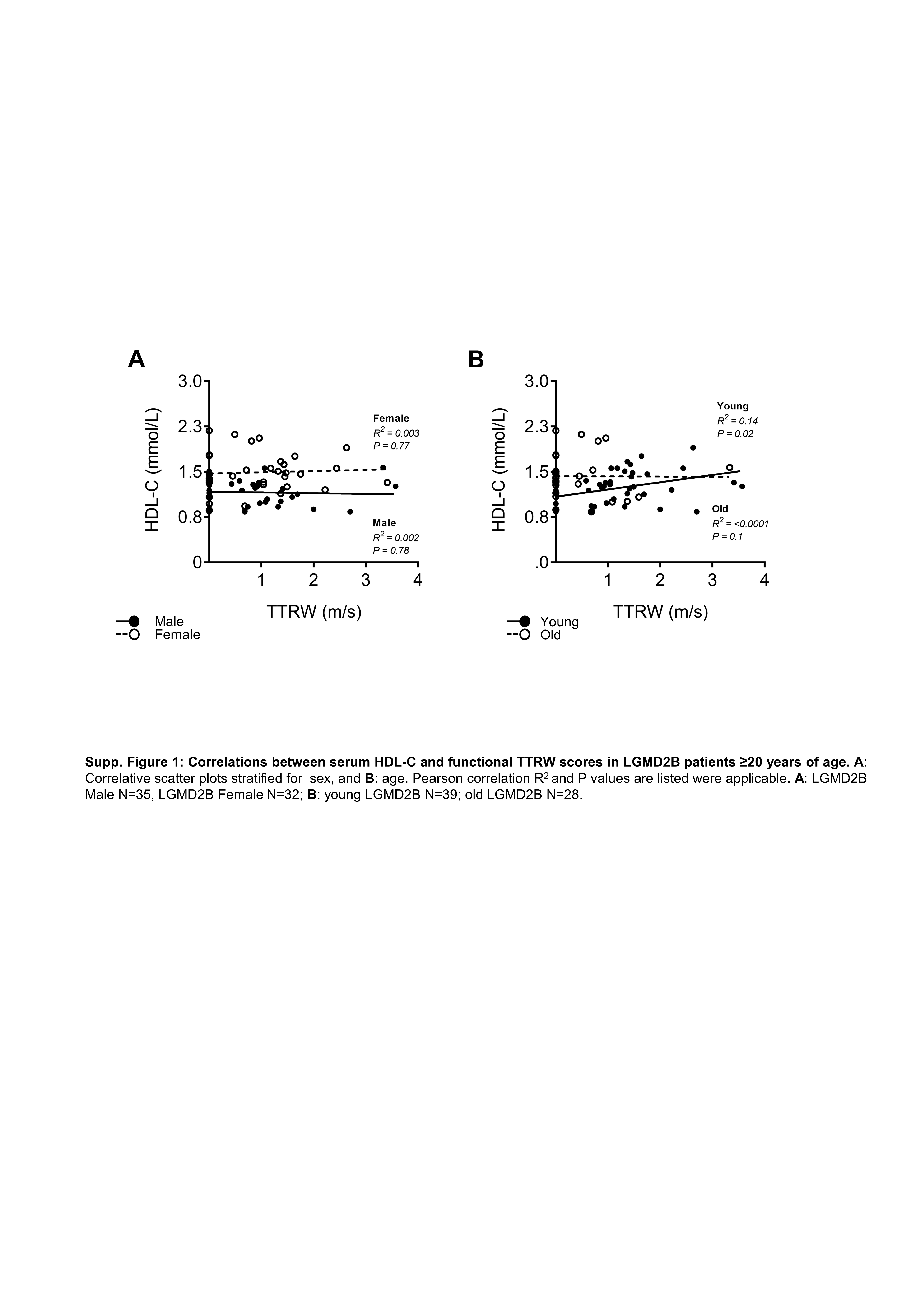

Supplement: Supplementary file 1 — Additional file 1: Supp. Figure 1. Correlations between serum HDL-C and functional TTRW scores in LGMD2B patients ≥20 years of age. A: Correlative scatter plots stratified for sex, and B: age. Pearson correlation R2 and P values are listed were applicable. A: LGMD2B Male N = 35, LGMD2B Female N = 32; B: young LGMD2B N = 39; old LGMD2B N = 28. [file 13395_2022_308_MOESM1_ESM.tif]

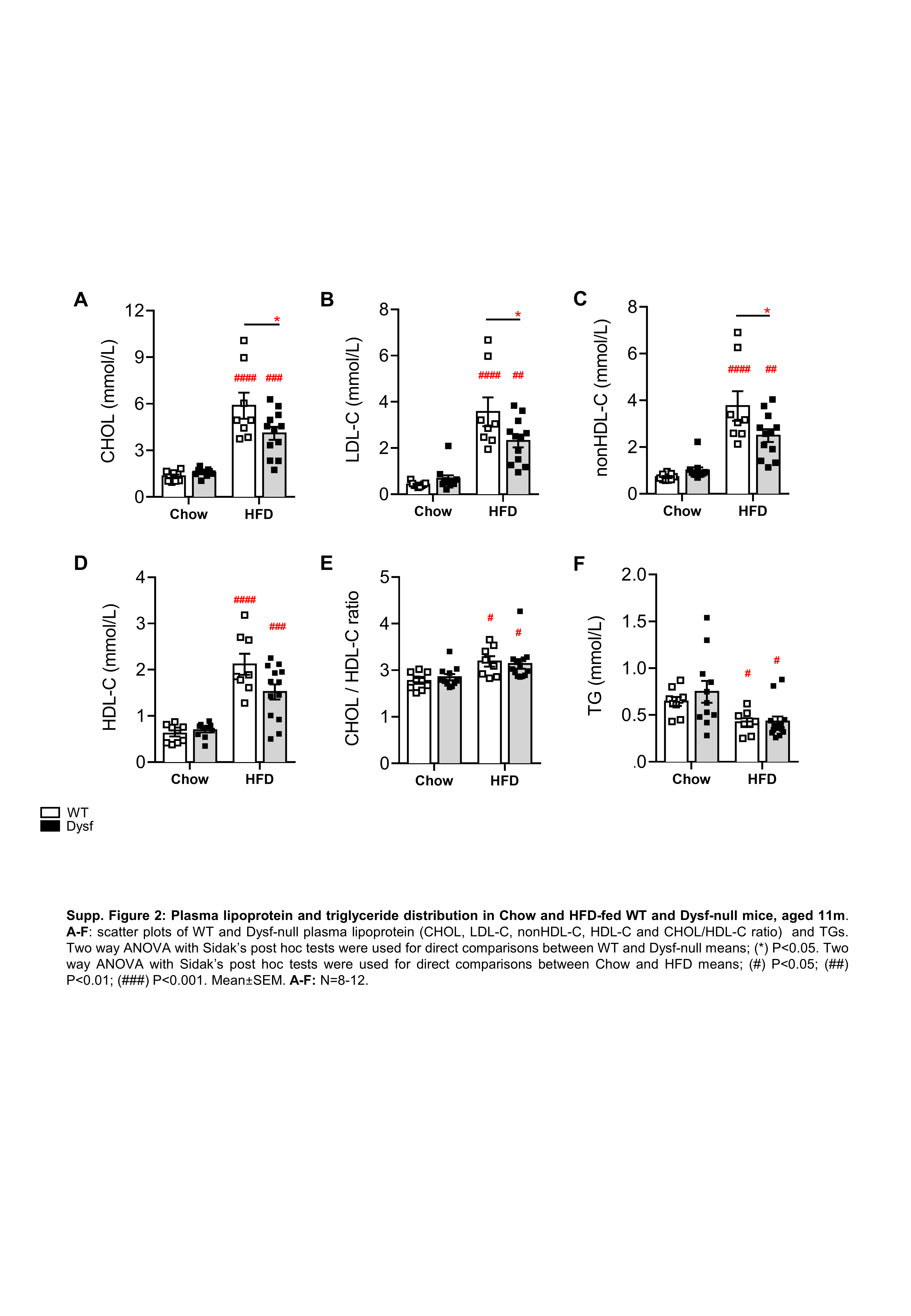

Supplement: Supplementary file 2 — Additional file 2: Supp. Figure 2. Plasma lipoprotein and triglyceride distribution in Chow and HFD-fed WT and Dysf-null mice, aged 11m. A-F: scatter plots of WT and Dysf-null plasma lipoprotein (CHOL, LDL-C, nonHDL-C, HDL-C and CHOL/HDL-C ratio) and TGs. Two way ANOVA with Sidak’s post hoc tests were used for direct comparisons between WT and Dysf-null means; (*) P < 0.05. Two way ANOVA with Sidak’s post hoc tests were used for direct comparisons between Chow and HFD means; (#) P < 0.05; (##) P < 0.01; (###) P < 0.001. Mean±SEM. A-F: N = 8-12. [file 13395_2022_308_MOESM2_ESM.tif]

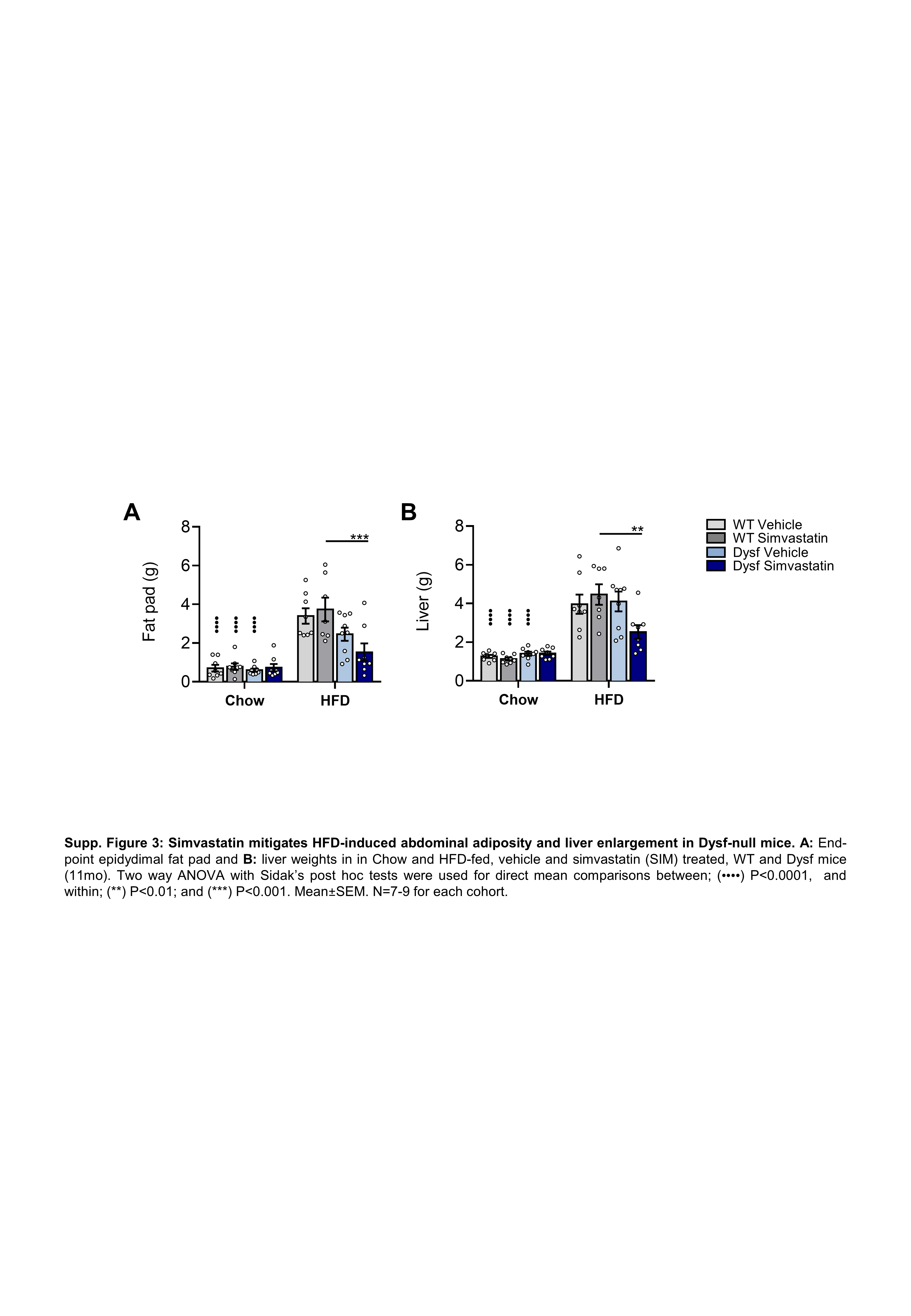

Supplement: Supplementary file 3 — Additional file 3: Supp. Figure 3. Simvastatin mitigates HFD-induced abdominal adiposity and liver enlargement in Dysf-null mice. A: End-point epidydimal fat pad and B: liver weights in in Chow and HFD-fed, vehicle and simvastatin (SIM) treated, WT and Dysf mice (11mo). Two way ANOVA with Sidak’s post hoc tests were used for direct mean comparisons between; (••••) P < 0.0001, and within; (**) P < 0.01; and (***) P < 0.001. Mean±SEM. N = 7-9 for each cohort. [file 13395_2022_308_MOESM3_ESM.tif]

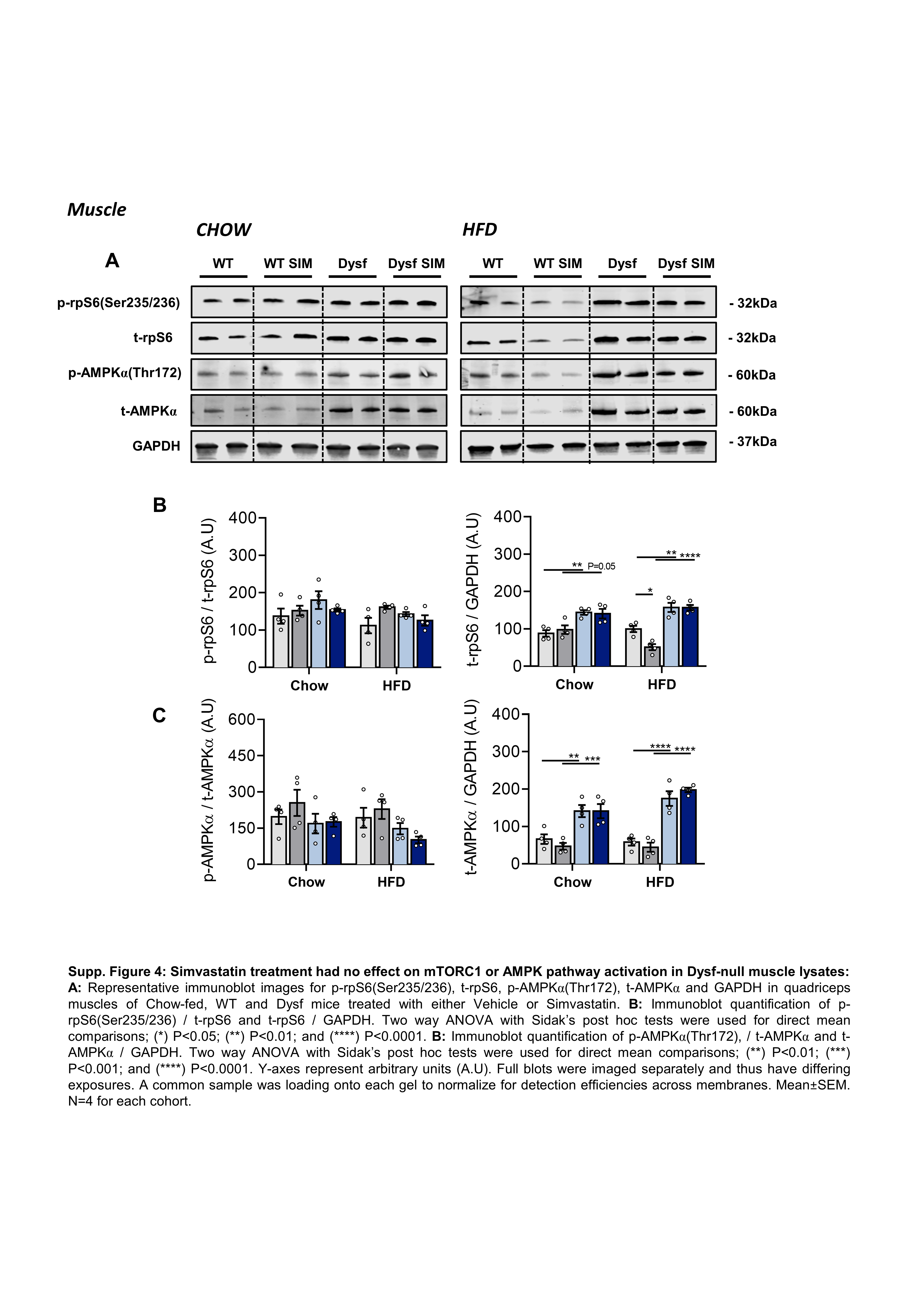

Supplement: Supplementary file 4 — Additional file 4: Supp. Figure 4. Simvastatin treatment had no effect on mTORC1 or AMPK pathway activation in Dysf-null muscle lysates: A: Representative immunoblot images for p-rpS6(Ser235/236), t-rpS6, p-AMPKα(Thr172), t-AMPKα and GAPDH in quadriceps muscles of Chow-fed, WT and Dysf mice treated with either Vehicle or Simvastatin. B: Immunoblot quantification of p-rpS6(Ser235/236) / t-rpS6 and t-rpS6 / GAPDH. Two way ANOVA with Sidak’s post hoc tests were used for direct mean comparisons; (*) P < 0.05; (**) P < 0.01; and (****) P < 0.0001. B: Immunoblot quantification of p-AMPKα(Thr172), / t-AMPKα and t-AMPKα / GAPDH. Two way ANOVA with Sidak’s post hoc tests were used for direct mean comparisons; (**) P < 0.01; (***) P < 0.001; and (****) P < 0.0001. Y-axes represent arbitrary units (A.U). Full blots were imaged separately and thus have differing exposures. A common sample was loading onto each gel to normalize for detection efficiencies across membranes. Mean±SEM. N = 4 for each cohort. [file 13395_2022_308_MOESM4_ESM.tif]
